# Optimistic Value Iteration

**DOI:** 10.1007/978-3-030-53291-8_26

**Published:** 2020-06-16

**Authors:** Arnd Hartmanns, Benjamin Lucien Kaminski

**Affiliations:** 8 Microsoft Research Lab, Redmond, WA USA; 9 University of Southern California, Los Angeles, CA USA; 10 University of Twente, Enschede, The Netherlands; 11 University College London, London, UK

## Abstract

Markov decision processes are widely used for planning and verification in settings that combine controllable or adversarial choices with probabilistic behaviour. The standard analysis algorithm, value iteration, only provides *lower bounds* on infinite-horizon probabilities and rewards. Two “sound” variations, which also deliver an *upper bound*, have recently appeared. In this paper, we present a new sound approach that leverages value iteration’s ability to *usually* deliver good lower bounds: we obtain a lower bound via standard value iteration, use the result to “guess” an upper bound, and prove the latter’s correctness. We present this *optimistic value iteration* approach for computing reachability probabilities as well as expected rewards. It is easy to implement and performs well, as we show via an extensive experimental evaluation using our implementation within the mcsta model checker of the Modest Toolset.

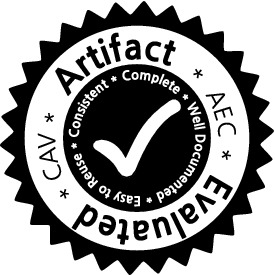



## Introduction

Markov decision processes (MDP, [30]) are a widely-used formalism to represent discrete-state and -time systems in which *probabilistic* effects meet controllable *nondeterministic* decisions. The former may arise from an environment or agent whose behaviour is only known statistically (e.g. message loss in wireless communication or statistical user profiles), or it may be intentional as part of a randomised algorithm (such as exponential backoff in Ethernet). The latter may be under the control of the system—then we are in a planning setting and typically look for a *scheduler* (or strategy, policy) that minimises the probability of unsafe behaviour or maximises a reward—or it may be considered adversarial, which is the standard assumption in verification: we want to establish that the maximum probability of unsafe behaviour is below, or that the minimum reward is above, a specified threshold. Extensions of MDP cover continuous time [11, 26], and the analysis of complex formalisms such as stochastic hybrid automata [13] can be reduced to the analysis of MDP abstractions.

The standard algorithm to compute optimal (maximum or minimum) probabilities or reward values on MDP is *value iteration* (VI). It implicitly computes the corresponding optimal scheduler, too. It keeps track of a value for every state of the MDP, locally improves the values iteratively until a “convergence” criterion is met, and then reports the final value for the initial state as the overall result. The initial values are chosen to be an underapproximation of the true values (e.g. 0 for all states in case of probabilities or non-negative rewards). The final values are then an improved underapproximation of the true values. For unbounded (infinite-horizon) properties, there is unfortunately no (known and practical) convergence criterion that could guarantee a predefined error on the final result. Still, probabilistic model checkers such as Prism [24] report the final result obtained via simple relative or absolute global error criteria as the definitive probability. This is because, on *most* case studies considered so far, value iteration in fact converges fast enough that the (relative or absolute) difference between the reported and the true value approximately meets the error $\epsilon$ specified for the convergence criterion. Only relatively recently has this problem of soundness come to the attention of the probabilistic verification and planning communities [7, 14, 28]. First highlighted on hand-crafted counterexamples, it has by now been found to affect benchmarks and real-life case studies, too [3].

The first proposal to compute sound reachability probabilities was to use *interval iteration* (II [15], first presented in [14]). The idea is to perform two iterations concurrently, one starting from 0 as before, and one starting from 1. The latter improves an overapproximation of the true values, and the process can be stopped once the (relative or absolute) difference between the two values for the initial state is below the specified $\epsilon$, or at any earlier time with a correspondingly larger but known error. Baier et al. extended interval iteration to expected accumulated reward values [3]; here, the complication is to find initial values that are guaranteed to be an overapproximation. The proposed graph-based (i.e. not numerical) algorithm in practice tends to compute conservative initial values from which many iterations are needed until convergence. More recently, *sound value iteration* (SVI) [31] improved upon interval iteration by computing upper bounds on-the-fly and performing larger value improvements per iteration, for both probabilities and expected rewards. However, we found SVI tricky to implement correctly; some edge cases not considered by the algorithm as presented in [31] initially caused our implementation to deliver incorrect results or diverge on very few benchmarks. Both II and SVI fundamentally depend on the MDP being *contracting*; this must be ensured by appropriate structural transformations, e.g. by collapsing end components, a priori. These transformations additionally complicate implementations, and increase memory requirements. *Our Contribution.* We present (in Sect. 4) a new algorithm to compute sound reachability probabilities and expected rewards that is both simple and practically efficient. We first (1) perform standard value iteration until “convergence”, resulting in a lower bound on the value for every state. To this we (2) apply specific heuristics to “guess”, for every state, a candidate upper bound value. Further value iterations (3) then confirm (if all values decrease) or disprove (if all values increase, or lower and upper bounds cross) the soundness of the upper bounds. In the latter case, we perform more lower bound iterations with reduced $\epsilon$ before retrying from step 2. We combine classic results from domain theory with specific properties of value iteration to show that our algorithm terminates. In problematic cases, many retries may be needed before termination, and performance may be worse than interval or sound value iteration. However, on many existing case studies, value iteration already worked well, and our approach attaches a soundness proof to its result with moderate overhead. We thus refer to it as *optimistic value iteration* (OVI). In contrast to II and SVI, it also works well for non-contracting MDP, albeit without a general termination guarantee. Our experimental evaluation in Sect. 5 uses all applicable models from the Quantitative Verification Benchmark Set [21] to confirm that OVI indeed performs as expected. It uses our publicly available implementations of II, SVI, and now OVI in the mcsta model checker of the Modest Toolset [20].

*Related Work.* In parallel to [15], the core idea behind II was also presented in [7] (later improved in [2]), embedded in a learning-based framework that manages to alleviate the state space explosion problem in models with a particular structure. In this approach, end components are statistically detected and collapsed on-the-fly. II has recently been extended to stochastic games in [23], offering *deflating* as a new alternative to collapsing end components in MDP. Deflating does not require a structural transformation, but rather extra computation steps in each iteration applied to the states of all (a priori identified) end components.

The only known convergence criterion for pure VI was presented in [9, Sect. 3.5]: if we run VI until the absolute error between two iterations is less than a certain value $\alpha$, then the computed values at that point are within $\alpha$ of the true values, and can in fact be rounded to the exact true values (as implemented in the *rational search* approach [5]). However, $\alpha$ cannot be freely chosen; it is a fixed number that depends on the size of the MDP and the largest denominator of the (rational) transition probabilities. The number of iterations needed is exponential in the size and the denominators. While not very useful in practice, this establishes an exponential upper bound on the number of iterations needed in unbounded-horizon VI. Additionally, Balaji et al. [4] recently showed the computations in finite-horizon value iteration to be EXPTIME-complete.

As an alternative to the iterative numeric road, guaranteed correct results (modulo implementation errors) can be obtained by using precise rational arithmetic. It does not combine too well with iterative methods like II or SVI due to the increasingly small differences between the values and the actual solution. The probabilistic model checker Storm [10] thus combines topological decomposition, policy iteration, and exact solvers for linear equation systems based on Gaussian elimination when asked to use rational arithmetic [22, Section 7.4.8]. The disadvantage is the significant runtime cost for performing the unlimited-precision calculations, limiting such methods to relatively smaller MDP.

The only experimental evaluations using large sets of benchmarks that we are aware of compared VI with II to study the overhead needed to obtain sound results via II [3], and II with SVI to show the performance improvements of SVI [31]. The learning-based method with deflation of [2] does not compete against II and SVI; its aim is rather in dealing with state space explosion (i.e. memory usage). Its performance was evaluated on 16 selected small (${<}400$ k states) benchmark instances in [2], showing absolute errors on the order of $10^{-4}$ on many benchmarks with a 30-min timeout. SVI thus appears the most competitive technique in runtime and precision so far. Consequently, in our evaluation in Sect. 5, we compare OVI with SVI, and II for reference, using the default relative error of $10^{-6}$, including large and excluding clearly acyclic benchmarks (since they are trivial even for VI), with a 10-min timeout which is rarely hit.

**Fig. 1.**
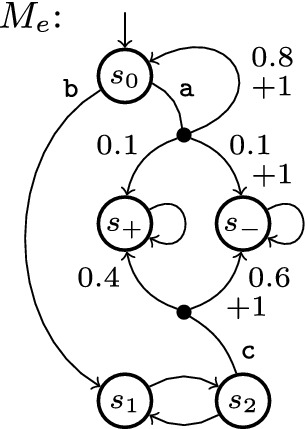
Example MDP

**Table 1.** VI and OVI example on $M_e$

*i*	$v(s_0)$	$u(s_0)$	$v(s_1)$	$u(s_1)$	$v(s_2)$	$u(s_2)$	$error$	$\alpha$
0	0		0		0			0.05
1	0.1		0		0.4		0.4	0.05
2	0.18		0.4		0.4		0.4	0.05
3	0.4		0.4		0.4		0.22	0.05
4	0.42	$\textit{0.47}$	0.4	$\textit{0.45}$	0.4	$\textit{0.45}$	0.02	0.05
5	0.436	$\overline{0.47}$	0.4	0.45	0.4	0.45	0.016	
6	0.4488		0.4		0.4		0.0128	0.008
7	0.45904		0.4		0.4		0.01024	0.008
8	0.467232		0.4		0.4		0.008192	0.008
9	0.4737856	$\textit{0.5237856}$	0.4	$\textit{0.45}$	0.4	$\textit{0.45}$	0.0065536	0.008
10	0.47902848	0.51902848	0.4	0.45	0.4	0.45	0.00524288	

## Preliminaries

$$\mathbb {R}^+_0 $$ is the set of all non-negative real numbers. We write $$\{\, x_1 \mapsto y_1, \dots \,\}$$ to denote the function that maps all $$x_i$$ to $$y_i$$, and if necessary in the respective context, implicitly maps to 0 all *x* for which no explicit mapping is specified. Given a set *S*, its powerset is $$2^{S} $$. A (discrete) *probability distribution* over *S* is a function $$\mu \in S \rightarrow [0, 1]$$ with countable *support*  and $$\sum _{s \in spt ({\mu })} \mu (s) = 1$$. $$ Dist ({S}) $$ is the set of all probability distributions over *S*.

*Markov Decision Processes* (MDP) combine nondeterministic choices as in labelled transition systems with discrete probabilistic decisions as in discrete-time Markov chains (DTMC). We define them formally and describe their semantics.

### Definition 1

A *Markov decision process* (MDP) is a triple $M =\langle S, s_I, T \rangle$ where *S* is a finite set of *states* with *initial state* $s_I \in S$ and $T : S \rightarrow 2^{ Dist ({\mathbb {R}^+_0 \times S})}$ is the *transition function*. *T*(*s*) must be finite and non-empty for all $s \in S$.

For $s \in S$, an element of *T*(*s*) is a *transition*, and a pair $\langle r, s' \rangle \in spt ({T(s)})$ is a *branch* to successor state $s'$ with *reward* *r* and probability $T(s)(\langle r, s' \rangle )$. Let $M^{(s_I')}$ be *M* but with initial state $s_I'$, and $M^0$ be *M* with all rewards set to zero.

### Example 1

Figure 1 shows our example MDP $M_e$. We draw transitions as lines to an intermediate node from which branches labelled with probability and reward (if not zero) lead to successor states. We omit the intermediate node and probability 1 for transitions with a single branch, and label some transitions to refer to them in the text. $M^e$ has 5 states, 7 transitions, and 10 branches.

In practice, higher-level modelling languages like Modest [17] are used to specify MDP. The semantics of an MDP is captured by its *paths*. A path represents a concrete resolution of all nondeterministic and probabilistic choices. Formally:

### Definition 2

A *finite path* is a sequence $\pi _\mathrm {fin} = s_0\, \mu _0\, r_0\, s_1\, \mu _1\, r_1 \dots \mu _{n-1} r_{n-1} s_n$ where $s_i \in S$ for all $i \in \{\, 0, \dots , n \,\}$ and $\exists \, \mu _i \in T(s_i) :\langle r_i, s_{i+1} \rangle \in spt ({\mu _i})$ for all $i \in \{\, 0, \dots , n - 1 \,\}$. Let , , and . $\varPi _ fin$ is the set of all finite paths starting in $s_I$. A *path* is an analogous infinite sequence $\pi$, and $\varPi$ is the set of all paths starting in $s_I$. We write $s \in \pi$ if $\exists \, i :s = s_i$, and $\pi _{\rightarrow G}$ for the shortest prefix of $\pi$ that contains a state in $G \subseteq S$, or $\bot$ if $\pi$ contains no such state. Let .

A scheduler (or *adversary*, *policy* or *strategy*) only resolves the nondeterministic choices of *M*. For this paper, memoryless deterministic schedulers suffice [6].

### Definition 3

A function $\mathfrak {s} :S \rightarrow Dist ({\mathbb {R}^+_0 \times S})$ is a *scheduler* if, for all $s \in S$, we have $\mathfrak {s}(s) \in T(s)$. The set of all schedulers of *M* is $\mathfrak {S}(M)$.

Given an MDP *M* as above, let $M|_\mathfrak {s} = \langle S, s_I, T|_\mathfrak {s} \rangle$ with $T|_\mathfrak {s}(s) = \{\,\mathfrak {s}(s)\,\}$ be the DTMC induced by $\mathfrak {s}$. Via the standard cylinder set construction [12, Sect. 2.2] on $M|_\mathfrak {s}$, a scheduler induces a probability measure $\mathbb {P}_\mathfrak {s}^M$ on measurable sets of paths starting in $s_I$. For goal state $g \in S$, the maximum and minimum **probability of reaching** $\textit{\textbf{g}}$ is defined as $\mathrm {P}_{\!\mathrm {max}}^M(\diamond \, g) = \sup _{\mathfrak {s} \in \mathfrak {S}} \mathbb {P}_\mathfrak {s}^M(\{\, \pi \in \varPi \mid g \in \pi \,\})$ and $\mathrm {P}_{\!\mathrm {min}}^M(\diamond \, g) = \inf _{\mathfrak {s} \in \mathfrak {S}} \mathbb {P}_\mathfrak {s}^M(\{\, \pi \in \varPi \mid g \in \pi \,\})$, respectively. The definition extends to sets *G* of goal states. Let $R_G^M :\varPi \rightarrow \mathbb {R}^+_0$ be the random variable defined by $R_G^M(\pi ) = \mathrm {rew}(\pi _{\rightarrow G})$ and let $\mathbb {E}_\mathfrak {s}^M(G)$ be the expected value of $R_G^M$ under $\mathbb {P}_\mathfrak {s}^M$. Then the maximum and minimum **expected reward to reach** $\textit{\textbf{G}}$ is defined as $\mathrm {E}_\mathrm {max}^M(G) = \sup _{\mathfrak {s}}\mathbb {E}_\mathfrak {s}^M(G)$ and $\mathrm {E}_\mathrm {min}^M(G) = \inf _{\mathfrak {s}}\mathbb {E}_\mathfrak {s}^M(G)$, respectively. We omit the superscripts for *M* when they are clear from the context. From now on, whenever we have an MDP with a set of goal states *G*, we assume that they have been made absorbing, i.e. for all $g \in G$ we only have a self-loop: $T(g) = \{\, \{\, \langle 0, g \rangle \mapsto 1 \,\} \,\}$.

### Definition 4

An *end component* of *M* as above is a (sub-)MDP $\langle S', T', s_I' \rangle$ where $S' \subseteq S$, $T'(s) \subseteq T(s)$ for all $s \in S'$, if $\mu \in T'(s)$ for some $s \in S'$ and $\langle r, s' \rangle \in spt ({\mu })$ then $r = 0$, and the directed graph with vertex set $S'$ and edge set $\{\, \langle s, s' \rangle \mid \exists \,\mu \in T'(s) :\langle 0, s' \rangle \in spt ({\mu }) \,\}$ is strongly connected.

## Value Iteration

The standard algorithm to compute reachability probabilities and expected rewards is *value iteration* (VI) [30]. In this section, we recall its theoretical foundations and its limitations regarding convergence.

### Theoretical Foundations

Let $\mathbb {V} = \{\,v ~|~ v :S \rightarrow \mathbb {R}^+_0 \cup \{\infty \}\,\}$ be a space of vectors of values. It can easily be shown that $\langle \mathbb {V},\, {\preceq } \rangle$ with

$$v \preceq w \qquad \text {if and only if}\qquad \forall \, s \in S:v(s) \le w(s)$$

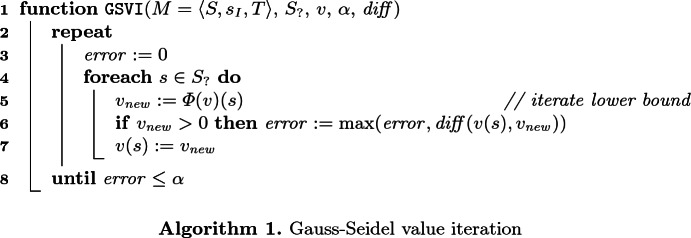


forms a complete lattice, i.e. every subset $V \subseteq \mathbb {V}$ has a supremum (and an infimum) in $\mathbb {V}$ with respect to $\preceq$. We write $v \prec w$ for $v \preceq w \wedge v \ne w$ and $v \not \sim w$ for $\lnot ( v \preceq w \vee w \preceq v )$.

Minimum and maximum reachability probabilities and expected rewards can be expressed as the *least fixed point* of the *Bellman operator* $\varPhi :\mathbb {V} \rightarrow \mathbb {V}$ given bywhere $opt \in \{\, \mathrm {max},\, \mathrm {min}\,\}$ and the choice of both $S_? \subseteq S$ and *d* depends on whether we wish to compute reachability probabilities or expected rewards. In any case, the Bellman operator $\varPhi$ can be shown to be Scott-continuous [1], i.e. in our case: for any subset $V \subseteq \mathbb {V}$, we have $\varPhi ( \sup V) = \sup \varPhi (V)$.

The Kleene fixed point theorem for Scott-continuous self-maps on complete lattices [1, 27] guarantees that $\textsf {lfp}\,\!\varPhi$, the least fixed point of $\varPhi$, indeed exists. Note that $\varPhi$ can still have more than one fixed point. In addition to mere existence of $\textsf {lfp}\,\varPhi$, the Kleene fixed point theorem states that $\textsf {lfp}\,\varPhi$ can be expressed by

1 $$\begin{aligned} \textsf {lfp}\,\varPhi = \lim _{n \rightarrow \infty } \varPhi ^n(\bar{0}) \end{aligned}$$

where $\bar{0} \in \mathbb {V}$ is the zero vector and $\varPhi ^n(v)$ denotes *n*-fold application of $\varPhi$ to *v*. Equation 1 is the basis of VI: the algorithm iteratively constructs a sequence of vectors

$$v_0 = \bar{0} \qquad \text {and}\qquad v_{i+1} = \varPhi (v_i),$$

which converges to the sought-after least fixed point. This convergence is *monotonic*: for every $n \in \mathbb {N}$, we have $\varPhi ^n(\bar{0}) \preceq \varPhi ^{n+1}(\bar{0})$ and hence $\varPhi ^n(\bar{0}) \preceq \textsf {lfp}\,\varPhi$. In particular, $\varPhi ^n(\bar{0})(s_I)$ is an *under*approximation of the sought-after quantity for every *n*. Note that iterating $\varPhi$ on *any* underapproximation $v \preceq \textsf {lfp}\,\varPhi$ (instead of $\bar{0}$) will still converge to $\textsf {lfp}\,\varPhi$ and $\varPhi ^n(v) \preceq \textsf {lfp}\,\varPhi$ will hold for any *n*.

*Gauss-Seidel Value Iteration.* Algorithm 1 shows the pseudocode of a VI implementation that uses the so-called *Gauss-Seidel optimisation*: Whereas standard VI needs to store two vectors $v_{i}$ and $v_{i+1}$, Gauss-Seidel VI stores only a single vector *v* and performs updates in place. This does not affect the correctness of VI, but may speed up convergence depending on the order in which the loop in line 4 considers the states in $S_?$. The error metric $diff$ is used to check for convergence.

*VI for Probabilities.* For determining reachability probabilities, we operate on $$M^0$$ and set $$S_? = S \setminus G$$ and $$d = 1$$. Then the corresponding Bellman operator satisfiesand VI will iteratively approximate this quantity *from below*. The corresponding call to Algorithm 1 is $$\texttt {GSVI}(M^0$$, $$S \setminus G$$, $$\{\, s \mapsto 0 \mid s \in S \setminus G \,\} \cup \{\, s \mapsto 1 \mid s \in G \,\}, \alpha $$, $$ diff )$$.

*VI for Expected Rewards.* For determining the expected reward $\mathrm {E}_{ opt }^{M^{(s)}}(G)$, we operate on *M* and first have to determine the set $S_\infty$ of states from which the minimum (if $opt = \mathrm {max}$) or maximum (if $opt = \mathrm {min}$) probability to reach *G* is less than 1.^1^ If $s_I \in S_\infty$, then the result is $\infty$ due to the definition of $\mathrm {rew}(\bot )$. Otherwise, we choose $S_? = S \setminus S_\infty$ and $d = \infty$. Then, for $opt = \mathrm {max}$, the least fixed point of the corresponding Bellman operator satisfies

$$(\textsf {lfp}\,\varPhi )(s) = \mathrm {E}_{ opt }^{M^{(s)}}(G).$$

Again, VI underapproximates this quantity. The same holds for $$ opt = \mathrm {min}$$ if *M* does not have end components containing states other than those in *G* and $$S_\infty $$. The corresponding call to Algorithm 1 is $$\texttt {GSVI}(M$$, $$S \setminus S_\infty $$, $$\{\, s \mapsto 0 \mid s \in S \setminus S_\infty \,\} \cup \{\, s \mapsto \infty \mid s \in S_\infty \,\}$$, $$\alpha $$, $$ diff )$$.

### Uniqueness of Fixed Points

$\textsf {lfp}\,\varPhi$ may not be unique for two reasons: states that cannot reach *G* under the optimal scheduler may take any value (causing fixed points greater than $\textsf {lfp}\,\varPhi$ for $\mathrm {P}_{\!\mathrm {min}}$ and $\mathrm {P}_{\!\mathrm {max}}$), and states in end components may take values higher than $\textsf {lfp}\,\varPhi$. The latter affects $\mathrm {P}_{\!\mathrm {max}}$ (higher fixed points) and $\mathrm {E}_\mathrm {min}$ (lower fixed points).

#### Example 2

In $M_e$ of Fig. 1, $s_1$ and $s_2$ and the two transitions in-between form an end component. For $\mathrm {P}_\mathrm {max}^{M_e}(\diamond \, \{\, s_+ \,\})$, $v = \{\, s \mapsto 1 \,\}$ is a non-least fixed point for the corresponding Bellman operator; with appropriate values for $s_1$ and $s_2$, we can obtain fixed points with any $v(s_0) > 0.5$ of our choice. Similarly, we have $\mathrm {E}_\mathrm {min}^M(\{\,s_+, s_-\,\}) = 0.6$ (by scheduling b in $s_0$), but due to the end component (with only zero-reward transitions by definition), the fixed point is s.t. $v(s_0) = 0$.

VI works for $\mathrm {P}_{\!\mathrm {min}}$, $\mathrm {P}_{\!\mathrm {max}}$, and $\mathrm {E}_\mathrm {max}$ with multiple fixed points: we anyway seek $\textsf {lfp}\,\varPhi$ and start from a (trivial) underapproximation. For $\mathrm {E}_\mathrm {min}$, (zero-reward) end components need to be collapsed: we determine the maximal end components using algorithms similar to [15, Alg. 1], then replace each of them by a single state, keeping all transitions leading out of the end component. We refer to this as the *ECC* transformation. However, such end components rarely occur in case studies for $\mathrm {E}_\mathrm {min}$ since they indicate Zeno behaviour w.r.t. to the reward. As rewards are often associated to time progress, such behaviour would be unrealistic.

To make the fixed points unique, for $\mathrm {E}_\mathrm {max}$ and $\mathrm {E}_\mathrm {min}$ we fix the values of all states in *G* to 0. For $\mathrm {P}_{\!\mathrm {min}}$, we precompute the set $S_\mathrm {min}^0$ of states that reach *G* with minimum probability 0 using Alg. 1 of [12], then fix their values to 0. For $\mathrm {P}_{\!\mathrm {max}}$, we analogously use $S_\mathrm {max}^0$ via Alg. 3 of [12]. For $\mathrm {P}_{\!\mathrm {max}}$ and $\mathrm {E}_\mathrm {min}$, we additionally need to remove end components via ECC. In contrast to the precomputations, ECC changes the structure of the MDP and is thus more memory-intensive.

### Convergence

VI and GSVI will not *reach* a fixed point in general, except for special cases such as acyclic MDP. It is thus standard to use a convergence criterion based on the difference between two consecutive iterations (lines 6 and 8) to make GSVI terminate: we either check the *absolute error*, i.e.or the *relative error*, i.e.By default, probabilistic model checkers like Prism and Storm use  and $\alpha = 10^{-6}$. Upon termination of GSVI, *v* is then closer to the least fixed point, but remains an underapproximation. In particular, $\alpha$ has, in general, no relation to the final difference between $v(s_I)$ and ${\mathrm {P}_{ \!\!opt }}(\diamond \, G)$ or ${\mathrm {E}_{ opt }}(G)$, respectively.

#### Example 3

Consider MDP $M_e$ of Fig. 1 again with $G = \{\, s_+ \,\}$. The first four rows in the body of Table 1 show the values for *v* after the *i*-th iteration of the outer loop of a call to . After the fourth iteration, GSVI terminates since the error is less than $\alpha = 0.05$; at this point, we have $\mathrm {P}_{\!\mathrm {max}}(\diamond \, s_+) - v(s_0) = 0.08 > \alpha$.

To obtain a value within a prescribed error $$\epsilon $$ of the true value, we can compute an upper bound in addition to the lower bound provided by VI. Interval iteration (II) [3, 15] does so by performing, in parallel, a second value iteration on a second vector *u* that starts from a known overapproximation. For probabilities, the vector $$\bar{1} = \{\,s \mapsto 1\,\}$$ is a trivial overapproximation; for rewards, more involved graph-based algorithms need to be used to precompute (a very conservative) one [3]. II terminates when $$ diff (v(s_I), u(s_I)) \le 2\epsilon $$ and returns $$v_{ II } = \frac{1}{2}(u(s_I) + v(s_I))$$. With , II thus guarantees that $$v_{ II } \in [v_ true - \epsilon \cdot v_ true , v_ true + \epsilon \cdot v_ true ]$$ and analogously for expected rewards. However, to ensure termination, II requires a unique fixed point: *u* converges from above to the greatest fixed point $$\textsf {gfp}\,\varPhi $$, thus for every MDP where $$ diff ((\textsf {lfp}\,\varPhi )(s_I), (\textsf {gfp}\,\varPhi )(s_I)) > 2\epsilon $$, II diverges. For $$\mathrm {P}_{\!\mathrm {max}}$$, we have $$\textsf {gfp}\,\varPhi (s_ ec ) = 1$$ for all $$s_ ec $$ in end components, thus II tends to diverge when there is an end component. Sound value iteration (SVI) [31] is similar, but uses a different approach to derive upper bounds that makes it perform better overall, and that eliminates the need to precompute an initial overapproximation for expected rewards. However, SVI still requires unique fixed points.

We summarise the preprocessing requirements of VI, II, and SVI in Table 2. With unique fixed points, we can transform $\mathrm {P}_{\!\mathrm {min}}$ into $\mathrm {P}_{\!\mathrm {max}}$ by making $S_\mathrm {min}^0$ states absorbing and setting *G* to $S_\mathrm {min}^0$, and $\mathrm {P}_{\!\mathrm {max}}$ into $\mathrm {E}_\mathrm {max}$ by a similar transformation adding reward 1 to entering *G*. Most of the literature on VI variants works in such a setting and describes the $\mathrm {P}_{\!\mathrm {max}}$ or $\mathrm {E}_\mathrm {max}$ case only. Since OVI also works with multiple fixed points, we have to consider all four cases individually.

**Table 2.** Preprocessing requirements of value iteration variants

Type	VI	II and SVI	OVI
$\mathrm {P}_{\!\mathrm {min}}$	–	$S_\mathrm {min}^0$	–
$\mathrm {P}_{\!\mathrm {max}}$	–	$S_\mathrm {max}^0 + \text {ECC}$	ECC$^{\text {a}}$
$\mathrm {E}_\mathrm {min}$	$S_\mathrm {max}^1 + \text {ECC}$	$S_\mathrm {max}^1 + \text {ECC}$	$S_\mathrm {max}^1 + \text {ECC}$
$\mathrm {E}_\mathrm {max}$	$S_\mathrm {min}^1$	$S_\mathrm {min}^1$	$S_\mathrm {min}^1$

$^{\text {a}}$ECC preprocessing for OVI is needed to guarantee termination in theory, however we have not yet found a case study where OVI diverges without ECC.

## Optimistic Value Iteration

We now present a new, practical solution to the convergence problem for unbounded reachability and expected rewards. It exploits the empirical observation that on many case studies VI delivers results which are roughly $\alpha$-close to the true value—it only lacks the ability to prove it. Our approach, *optimistic value iteration* (OVI), extends standard VI with the ability to deliver such a proof.

The key idea is to exploit a property of the Bellman operator $\varPhi$ and its Gauss-Seidel variant as in Algorithm 1 to determine whether a candidate vector is a lower bound, an upper bound, or neither. The foundation is basic domain theory: by Scott-continuity of $\varPhi$ it follows that $\varPhi$ is monotonic, meaning $v \preceq w$ implies $\varPhi (v) \preceq \varPhi (w)$. A principle called *Park induction* [29] for monotonic self-maps on complete lattices yields the following induction rules: For any $u \in \mathbb {V}$,

2 $$\begin{aligned}&\varPhi (u) \preceq u \qquad \text {implies}\qquad \textsf {lfp}\,\varPhi \preceq u. \end{aligned}$$

3 $$\begin{aligned} \text {and} \qquad&u \preceq \varPhi (u) \qquad \text {implies}\qquad u \preceq \textsf {gfp}\,\varPhi .\qquad \end{aligned}$$

Thus, if we can construct a candidate vector *u* s.t. $\varPhi (u) \preceq u$, then *u* is in fact an upper bound on the sought-after $\textsf {lfp}\,\!{\varPhi }$. We call such a *u* an *inductive upper bound*. Optimistic value iteration uses this insight and can be summarised as follows:
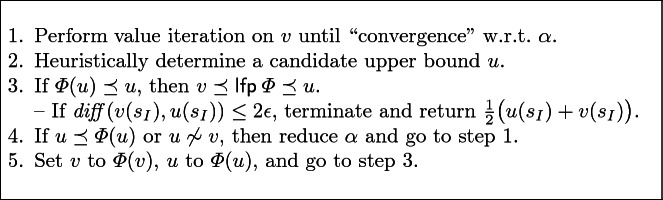
 
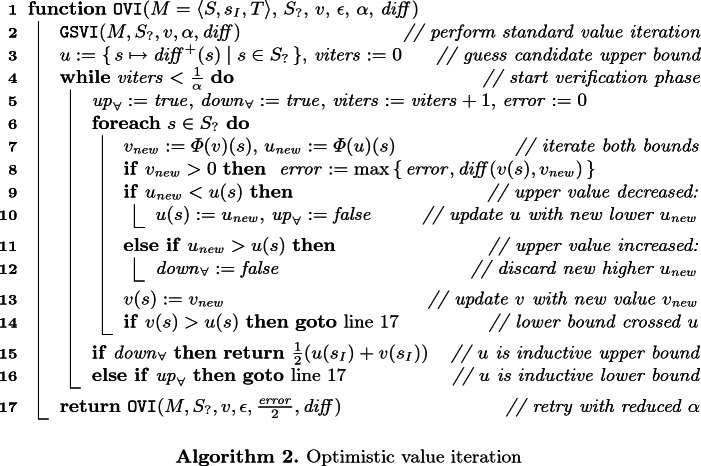


The resulting procedure in more detail is shown as Algorithm 2. Starting from the same initial vectors *v* as for VI, we first perform standard Gauss-Seidel value iteration (in line 2). We refer to this as the *iteration phase* of OVI. After that, vector *v* is an improved underapproximation of the actual probabilities or reward values. We then “guess” a vector *u* of *upper values* from the *lower values* in *v* (line 3). The guessing heuristics depends on $diff$: if , then we useif , then

$$ diff ^+(s) = v(s) \cdot (1 + \epsilon ).$$

We cap the result at 1 for $$\mathrm {P}_{\!\mathrm {min}}$$ and $$\mathrm {P}_{\!\mathrm {max}}$$. These heuristics have three important properties: (**H1**) $$v(s) = 0$$ implies $$ diff ^+(s) = 0$$, (**H2**) $$ diff (v(s), diff ^+(s)) \le 2\epsilon $$, and (**H3**) $$ diff (v(s), diff ^+(s)) > 0$$ unless $$v(s) = 0$$ or $$v(s) = 1$$ for $$\mathrm {P}_{\!\mathrm {min}}$$ and $$\mathrm {P}_{\!\mathrm {max}}$$.

Then the *verification phase* starts in line 4: we perform value iteration on the lower values *v* and upper values *u* at the same time, keeping track of the direction in which the upper values move. For *u*, line 7 and the conditions around line 10 mean that we actually use operator $\varPhi _\mathrm {min}(u) = \lambda \,s.~\mathrm {min}( \varPhi (u)(s), u(s) )$. This may shorten the verification phases, and is crucial for our termination argument. A state *s* is *blocked* if $\varPhi (u)(s) > \varPhi _\mathrm {min}(u)(s)$ and *unblocked* if $\varPhi (u)(s) < u(s)$ here.

If, in some iteration, no state was blocked (line 15), then we had $\varPhi (u) \preceq u$ before the start of the iteration. We thus know by Eq. 2 that the current *u* is an inductive upper bound for the values of all states, and the true value must be in the interval $[v(s_I), u(s_I)]$. By property H2, our use of $\varPhi _\mathrm {min}$ for *u*, and the monotonicity of $\varPhi$ as used on *v*, we also know that $diff (v(s_I), u(s_I)) \le 2\epsilon$, so we immediately terminate and return the interval’s centre $v_I = \frac{1}{2}(u(s_I) + v(s_I))$. The true value $v_ true = (\textsf {lfp}\,\varPhi )(s_I)$ must then be in $[v_I - \epsilon \cdot v_ true , v_I + \epsilon \cdot v_ true ]$.

If, in some iteration, no state was unblocked (line 16), then again by Park induction we know that $u \preceq \textsf {gfp}\,\varPhi$. If we are in a situation of unique fixed points, this also means $u \preceq \textsf {lfp}\,\varPhi$, thus the current *u* is no upper bound: we cancel verification and go back to the iteration phase to further improve *v* before trying again. We do the same if *v* crosses *u*: then $u(s) < v(s) \le (\textsf {lfp}\,\varPhi )(s)$ for some *s*, so this *u* was just another bad guess, too.

Otherwise, we do not yet know the relationship between *u* and $\textsf {lfp}\,\varPhi$, so we remain in the verification phase until we encounter one of the cases above, or until we exceed the verification budget of $\frac{1}{\alpha }$ iterations (as checked by the loop condition in line 4). This budget is a technical measure to ensure termination.

*Optimisation.* In case the fixed point of $\varPhi$ is *unique*, by Park induction (via Eq. 3) we know that $u \preceq \varPhi (u)$ implies that *u* is a lower bound on $\textsf {lfp}\,\varPhi$. In such situations of single fixed points, we can—as an optimisation—additionally replace *v* by *u* before the *goto* in line 16.

*Heuristics.* OVI relies on heuristics to gain an advantage over alternative methods such as II or SVI; it cannot be better on *all* MDP. Concretely, we can choose

1. a stopping criterion for the iteration phase,
2. how to guess candidate upper values from the result of the iteration phase, and
3. how much to reduce $\alpha$ when going back from verification to iteration.

Algorithm 2 shows the choices made by our implementation. We employ the standard stopping criteria used by probabilistic model checkers for VI, and the “weakest” guessing heuristics that satisfies properties H1, H2, and H3 (i.e. guessing any higher values would violate one of these properties). The only arbitrary choice is how to reduce $\alpha$, which we at least halve on every retry. We experimentally found this to be a good compromise on benchmarks that we consider in Sect. 5, where

- reducing $\alpha$ further causes more and potentially unnecessary iterations in GSVI (continuing to iterate when switching to the verification phase would already result in upper values sufficient for termination), and
- reducing $\alpha$ less results in more verification phases (whose iterations are computationally more expensive than those of GSVI) being started before the values in *v* are high enough such that we manage to guess a *u* with $\textsf {lfp}\,\varPhi \preceq u$.

### Example 4

We now use the version of $\varPhi$ to compute $\mathrm {P}_{\!\mathrm {max}}$ and callTable 1 shows the values in *v* and *u* during this run, assuming that we use non-Gauss-Seidel iterations. The first iteration phase lasts from $i = 0$ to 4. At this point, *u* is initialised with the values shown in italics. The first verification phase needs only one iteration to realise that *u* is actually a lower bound (to a fixed point which is not the least fixed point, due to the uncollapsed end component). Blocked states are marked with a $\overline{\text {bar}}$; unblocked states have a lower *u*-value than in the previous iteration. We resume GSVI from $i = 6$. The error in GSVI is again below $\alpha$, which had been reduced to 0.008, during iteration $i = 9$. We thus start another verification phase, which immediately (in one iteration) finds the newly guessed vector *u* to be an upper bound, with $diff (v(s_0), u(s_0)) < 2\epsilon$.

### Termination of OVI

We showed above that OVI returns an $\epsilon$-correct result when it terminates. We now show that it terminates in all cases except for $\mathrm {P}_{\!\mathrm {max}}$ with multiple fixed points. Note that this is a stronger result than what II and SVI can achieve.

Let us first consider the situations where $\textsf {lfp}\,\varPhi$ is the unique fixed point of $\varPhi$. First, GSVI terminates by Eq. 1. Let us now write $v_i$ and $u_i$ for the vectors *u* and *v* as they are at the beginning of verification phase iteration *i*. We know that $v_0 \preceq u_0$. We distinguish three cases relating the initial guess $u_0$ to $\textsf {lfp}\,\varPhi$.

1. $u_0 \not \sim \textsf {lfp}\,\varPhi$ or $u_0 \prec \textsf {lfp}\,\varPhi$, i.e. there is a state *s* with $u_0(s) < (\textsf {lfp}\,\varPhi )(s)$. Since we use $\varPhi _\mathrm {min}$ on the upper values, it follows $u_i(s) \le u_0(s) < (\textsf {lfp}\,\varPhi )(s)$ for all *i*. By Eq. 1, there must thus be a *j* such that $v_j(s) > u_j(s)$, triggering a retry with reduced $\alpha$ in line 14. Such a retry could also be triggered earlier in line 16. Due to the reduction of $\alpha$ and Eq. 1, every call to GSVI will further increase some values in *v* or reach $v = \textsf {lfp}\,\varPhi$ (in special cases), and for some subsequent guess *u* we must have $u_0(s) < u(s)$. Consequently, after some repetitions of this case 1, we must eventually guess a *u* with $\textsf {lfp}\,\varPhi \preceq u$.
2. $\textsf {lfp}\,\varPhi \prec u_0$. Observe that operators $\varPhi$ and $\varPhi _\mathrm {min}$ are *local* [9], i.e. a state’s value can only change if a direct successor’s value changes. In particular, a state’s value can only decrease (increase) if a direct successor’s value decreases (increases). If $u_i(s) < u_{i-1}(s)$, then *s* cannot be blocked again in any later iteration $j > i$: for it to become blocked, a successor’s upper value would have to increase, but $\varPhi _\mathrm {min}$ ensures non-increasing upper values for all states. Analogously to Eq. 1, we know that [3, Lemma 3.3 (c)]
  $$ \textsf {lfp}\,\varPhi \preceq u \quad \text {implies}\quad \lim _{n\rightarrow \infty } \varPhi _\mathrm {min}^n(u) = \textsf {lfp}\,\varPhi $$
  (for the unique fixpoint case, since [3] assumes contracting MDP as usual). Thus, for all states *s*, there must be an *i* such that $u_i(s) < u_{i-1}(s)$; in consequence, there is also an iteration *j* where no state is blocked any more. Then the condition in line 15 will be true and OVI terminates.
3. $\textsf {lfp}\,\varPhi \preceq u_0$ but not $\textsf {lfp}\,\varPhi \prec u_0$, i.e. there is a state *s* with $u_0(s) = (\textsf {lfp}\,\varPhi )(s)$. If there is an *i* where no state, including *s*, is blocked, then OVI terminates as above. For $\mathrm {P}_{\!\mathrm {min}}$ and $\mathrm {P}_{\!\mathrm {max}}$, if $u_0(s) = 1$, *s* cannot be blocked, so we can w.l.o.g. exclude such *s*. For other *s* not to be blocked in iteration *i*, we must have $u_i(s') = (\textsf {lfp}\,\varPhi )(s')$ for all states $s'$ reachable from *s* under the optimal scheduler, i.e. all of those states must *reach* the fixed point. This cannot be guaranteed on general MDP. Since this case is a very particular situation unlikely to be encountered in practice with our heuristics, OVI adopts a pragmatic solution: it bounds the number of iterations in every verification phase (cf. line 4). Due to property H3 of our heuristics, $u_0(s) = (\textsf {lfp}\,\varPhi )(s)$ requires $v_0(s) < (\textsf {lfp}\,\varPhi )(s)$, thus some subsequent guess *u* will have $u(s) > u_0(s)$, and eventually we must get a *u* with $\textsf {lfp}\,\varPhi \prec u$, which is case 2. Since we strictly increase the iteration bound on every retry, we will eventually encounter case 2 with a sufficiently high bound for termination.

Three of the four situations with multiple fixed points reduce to the corresponding unique fixed point situation due to property H1 of our guessing heuristics:

1. For $\mathrm {P}_{\!\mathrm {min}}$, recall from Sect. 3.2 that the fixed point is unique if we fix the values of all $S_\mathrm {min}^0$ states to 0. In OVI without preprocessing, such states are in $S_?$, thus they initially have value 0. $\varPhi$ will not increase their values, neither will guessing due to H1, and neither will $\varPhi _\mathrm {min}$. Thus OVI here operates on a sublattice of $\langle \mathbb {V},\, {\preceq } \rangle$ where the fixed point of $\varPhi$ is unique.
2. For $\mathrm {E}_\mathrm {min}$, after the preprocessing steps of Table 2, we only need to fix the values of all goal states to 0. Then the argument is the same as for $\mathrm {P}_{\!\mathrm {min}}$.
3. For $\mathrm {E}_\mathrm {max}$, we reduce to a unique fixed point sublattice in the same way, too.

The only case where OVI may not terminate is for $\mathrm {P}_{\!\mathrm {max}}$ without ECC. Here, end components may cause states to be permanently blocked. However, we did not encounter this on any benchmark used in Sect. 5, so in contrast to e.g. II, OVI is still *practically* useful in this case despite the lack of a termination guarantee.

#### Example 5

We turn $M_e$ of Fig. 1 into $M_e'$ by replacing the c-labelled transition from $s_2$ by transition $\{\, \langle 0, s_2 \rangle \mapsto \frac{1}{2}, \langle 0, s_+ \rangle \mapsto \frac{1}{4}, \langle 1, s_- \rangle \mapsto \frac{1}{4} \,\}$, i.e. we can now go from $s_2$ back to $s_2$ with probability $\frac{1}{2}$ and to each of $s_+$, $s_-$ with probability $\frac{1}{4}$. The probability-1 transition from $s_2$ to $s_1$ remains. Then Table 3 shows a run of OVI for $\mathrm {P}_{\!\mathrm {max}}$ with  and $\alpha = 0.1$. $s_0$ is forever blocked from iteration 6 on.

**Fig. 2.**
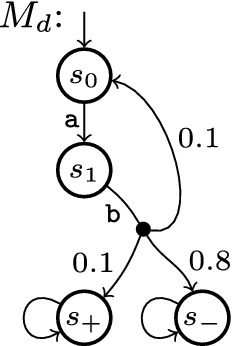
DTMC $$M_d$$

**Table 3.** Nontermination of OVI on $M_e'$ without ECC

*i*	$v(s_0)$	$u(s_0)$	$v(s_1)$	$u(s_1)$	$v(s_2)$	$u(s_2)$	$error$	$\alpha$
0	0		0		0			0.05
1	0.1		0		0.25		0.25	0.05
2	0.18		0.25		0.375		0.25	0.05
3	0.25		0.375		0.4375		0.125	0.05
4	0.375		0.4375		0.46875		0.125	0.05
5	0.4375	$\textit{0.5375}$	0.46875	$\textit{0.56875}$	0.484375	$\textit{0.584375}$	0.0625	0.05
6	0.46875	$\overline{0.5375}$	0.484375	$\overline{0.56875}$	0.4921875	0.56875	0.03125	
7	0.484375	$\overline{0.5375}$	0.4921875	0.56875	0.49609375	0.56875	0.015625	

### Variants of OVI

While the core idea of OVI rests on classic results from domain theory, Algorithm 2 includes several particular choices that work together to achieve good performance and ensure termination. We sketch two variants to motivate these choices.

First, let us use $$\varPhi $$ instead of $$\varPhi _\mathrm {min}$$ for the upper values, i.e. move the assignment $$u(s) := u_ new $$ down into line 13. Then we cannot prove termination because the arguments of case 2 for $$\textsf {lfp}\,\varPhi \prec u_0$$ no longer hold. Consider DTMC $$M_d$$ of Fig. 2 and $$\mathrm {P}_{\!\mathrm {max}}(\diamond \, s_+) = \mathrm {P}_{\!\mathrm {min}}(\diamond \, s_+)$$. Let

$$ u = \{\, s_0 \mapsto 0.2, s_1 \mapsto 1, s_+ \mapsto 1, s_- \mapsto 0 \,\} \succ \textstyle \{\, s_0 \mapsto \frac{1}{9}, s_1 \mapsto \frac{1}{9}, \dots \,\} = \textsf {lfp}\,\varPhi . $$

Iterating $\varPhi$, we then get the following sequence of pairs $\langle u(s_0), u(s_1) \rangle$:

$$ \langle 0.2, 1 \rangle , \langle 1, 0.12 \rangle , \langle 0.12, 0.2 \rangle , \langle 0.2, 0.112 \rangle , \langle 0.112, 0.12 \rangle , \langle 0.12, 0.1112 \rangle , \dots $$

Observe how the value of $s_0$ increases iff $s_1$ decreases and vice-versa. Thus we never encounter an inductive upper or lower bound. In Algorithm 2, we use Gauss-Seidel VI, which would not show the same effect on this model; however, if we insert another state between $s_0$ and $s_1$ that is updated last, Algorithm 2 would behave in the same alternating way. This particular *u* is contrived, but we could have guessed one with a similar relationship of the values leading to similar behaviour.

An alternative that allows us to use $\varPhi$ instead of $\varPhi _\mathrm {min}$ is to change the conditions that lead to retrying and termination: We separately store the initial guess of a verification phase as $u_0$, and then compare each newly calculated *u* with $u_0$. If $u \preceq u_0$, then we know that there is an *i* such that $u = \varPhi ^i(u) \preceq u_0$. $\varPhi ^i$ retains all properties of $\varPhi$ needed for Park induction, so this would also be a proof of $\textsf {lfp}\,\varPhi \preceq u$. The other conditions and the termination proofs can be adapted analogously. However, this variant needs $\approx$50 % more memory (to store an additional vector of values), and we found it to be significantly slower than Algorthm 2 and the first variant on almost all benchmark instances of Sect. 5.

## Experimental Evaluation

We have implemented interval iteration (II) (using the “variant 2” approach of [3] to compute initial overapproximations for expected rewards), sound value iteration (SVI), and now optimistic value iteration (OVI) precisely as described in the previous section, in the mcsta model checker of the Modest Toolset [20], which is publicly available at modestchecker.net. It is cross-platform, implemented in C#, and built around the Modest [17] high-level modelling language. Via support for the Jani format [8], mcsta can exchange models with other tools like Epmc [18] and Storm [10]. Its performance is competitive with Storm and Prism [16]. We tried to spend equal effort performance-tuning our VI, II, SVI, and OVI implementations to avoid unfairly comparing highly-optimised OVI code with naïve implementations of the competing algorithms.

In the following, we report on our experimental evaluation of OVI using mcsta on all applicable models of the Quantitative Verification Benchmark Set (QVBS) [21]. All models in the QVBS are available in Jani and can thus be used by mcsta. Most are parameterised, and come with multiple properties of different types. Aside from MDP models, the QVBS also includes DTMCs (which are a special case of MDP), continuous-time Markov chains (CTMC, for which the analysis of unbounded properties reduces to checking the embedded DTMC), Markov automata (MA [11], on which the embedded MDP suffices for unbounded properties), and probabilistic timed automata (PTA [26], some of which can be converted into MDP via the digital clocks semantics [25]). We use all of these model types. The QVBS thus gives rise to a large number of benchmark *instances*: combinations of a model, a parameter valuation, and a property to check. For every model, we chose one instance per probabilistic reachability and expected-reward property such that state space exploration did not run out of memory and VI took at least 10 s where possible. We only excluded

- 2 models with multiple initial states (which mcsta does not yet support),
- 4 PTA with open clock constraints (they cannot be converted to MDP),
- 29 probabilistic reachability properties for which the result is 0 or 1 (they are easily solved by the graph-based precomputations and do not challenge VI),
- 16 instances for which VI very quickly *reaches* the fixed point, which indicates that (the relevant part of) the MDP is acyclic and thus trivial to solve,
- 3 models for which no parameter valuation allowed state space exploration to complete without running out of memory or taking more than 600 s,
- 7 instances where, on the largest state space we could explore, no iterative algorithm took more than 1 s (which does not allow reliable comparisons), and
- the *oscillators* model due to its very large model files,

As a result, we considered 38 instances with probabilistic reachability and 41 instances with expected-reward properties, many comprising several million states.

**Fig. 3.**
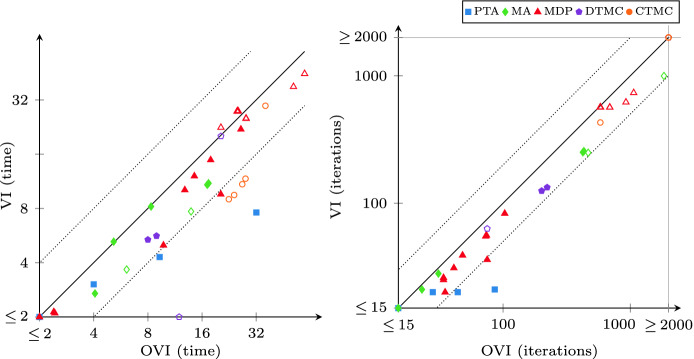
OVI runtime and iteration count compared to VI (probabilistic reachability)

We ran all experiments on an Intel Core i7-4790 workstation (3.6–4.0  GHz) with 8 GB of memory and 64-bit Ubuntu Linux 18.04. By default, we request a relative half-width of $\epsilon = 10^{-6}$ for the result probability or reward value, and configure OVI to use the relative-error criterion with $\alpha = 10^{-6}$ in the iteration phase. We use a 600 s timeout (“TO”). Due to the number of instances, we show most results as scatter plots like in Fig. 3. Each such plot compares two methods in terms of runtime or number of iterations. Every point $\langle x, y \rangle$ corresponds to an instance and indicates that the method noted on the x-axis took *x* seconds or iterations to solve this instance while the method noted on the y-axis took *y* seconds or iterations. Thus points above the solid diagonal line correspond to instances where the x-axis method was faster (or needed fewer iterations); points above (below) the upper (lower) dotted diagonal line are where the x-axis method took less than half (more than twice) as long or as many iterations.

**Fig. 4.**
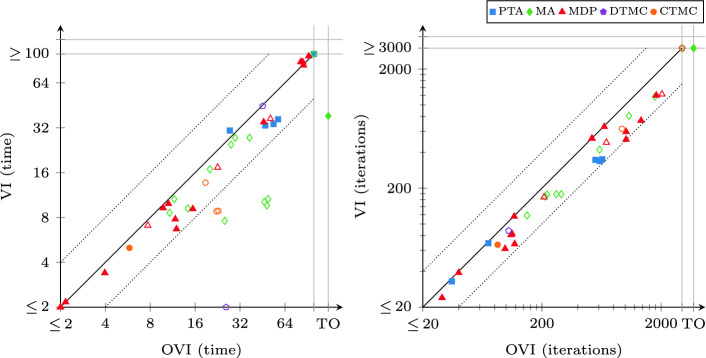
OVI runtime and iteration count compared to VI (expected rewards)

**Fig. 5.**
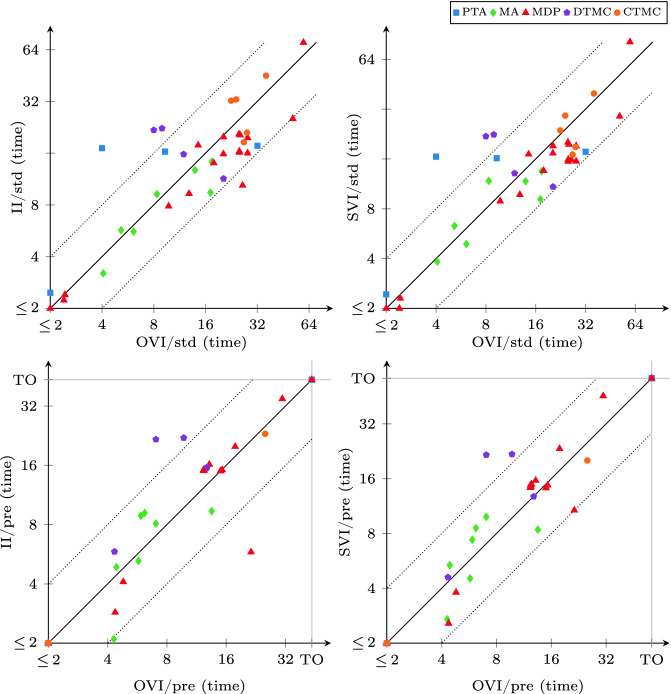
OVI runtime compared to II and SVI (probabilities)

### Comparison with VI

All methods except VI delivered correct results up to $\epsilon$. VI offers low runtime at the cost of occasional incorrect results, and in general the absence of any guarantee about the result. We thus compare with VI separately to judge the overhead caused by performing additional verification, and possibly iteration, phases. This is similar to the comparison done for II in [3]. Figures 3 and 4 show the results. The unfilled shapes indicate instances where VI produced an incorrect result. In terms of runtime, we see that OVI does not often take more than twice as long as VI, and frequently requires less than 50% extra time. On several instances where OVI incurs most overhead, VI produces an incorrect result, indicating that they are “hard” instances for value iteration. The unfilled CTMCs where OVI takes much longer to compute probabilities are all instances of the *embedded* model; the DTMC on the x-axis is *haddad-monmege*, an adversarial model built to highlight the convergence problem of VI in [14]. The problematic cases for expected rewards include most MA instances, the two expected-reward instances of the *embedded* CTMC, and again *haddad-monmege*. In terms of iterations, the overhead of OVI is even less than in runtime.

**Fig. 6.**
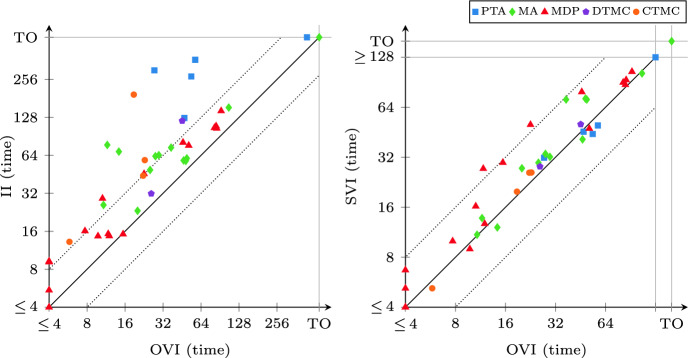
OVI runtime compared to II and SVI (expected rewards)

### Comparison with II and SVI

We compare the runtime of OVI with the runtime of II and that of SVI separately for reachability probabilities (shown in Fig. 5) and expected rewards (shown in Fig. 6). As shown in Table 2, OVI has almost the same requirements on precomputations as VI, while II and SVI require extra precomputations and ECC for reachability probabilities. The precomputations and ECC need extra runtime (which turned out to be negligible in some cases but significant enough to cause a timeout in others) prior to the numeric iterations. However, doing the precomputations can reduce the size of the set $S_?$, and ECC can reduce the size of the MDP itself. Both can thus reduce the runtime needed for the numeric iterations. For the overall runtime, we found that none of these effects dominates the other over all models. Thus sometimes it may be better to perform only the required precomputations and transformations, while on other models performing all applicable ones may lead to lower total runtime. For reachability probabilities, we thus compare OVI, II, and SVI in two scenarios: once in the default (“std”) setting of mcsta that uses only required preprocessing steps (without ECC for OVI; we report the total runtime for preprocessing and iterations), and once with all of them enabled (“pre”, where we report only the runtime for numeric iterations, plus the computation of initial upper bounds in case of II).

For probabilistic reachability, we see in Fig. 5 that there is no clear winner among the three methods in the “std” setting (top plots). In some cases, the extra precomputations take long enough to give an advantage to OVI, while in others they speed up II and SVI significantly, compensating for their overhead. The “pre” setting (bottom), in which all three algorithms operate on exactly the same input w.r.t. to MDP *M* and set $S_?$, however, shows a clearer picture: now OVI is faster, sometimes significantly so, than II and SVI on most instances.

Expected-reward properties were more challenging for all three methods (as well as for VI, which produced more errors here than for probabilities). The plots in Fig. 6 paint a very clear picture of OVI being significantly faster for expected rewards than II (which suffers from the need to precompute initial upper bounds that then turn out to be rather conservative), and faster (though by a lesser margin and with few exceptions) than SVI.

**Fig. 7.**
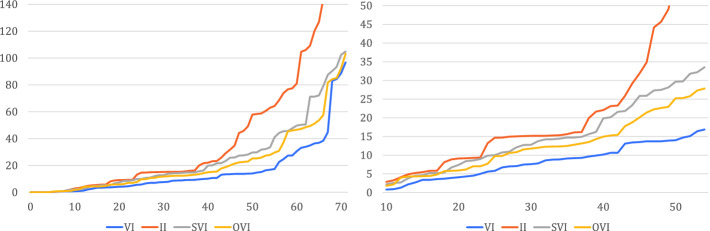
Summary comparison to VI, II, and SVI, instances ordered by runtime

In Fig. 7, we give a summary view combining the data from Figs. 3 to 6. For each algorithm, we plot the instances sorted by runtime, i.e. a point $\langle x, y \rangle$ on the line for algorithm *z* means that some instance took *y* seconds to solve via *z*, and there are *x* instances that *z* solves in less time. Note in particular that the times are *not* cumulative. The right-hand plot zooms into the left-hand one. We clearly see the speedup offered by OVI over SVI and especially II. Where the scatter plots merely show that OVI often does not obtain more than a $2\times$ speedup compared to SVI, these plots provide an explanation: the VI line is a rough bound on the performance that any *extension* of VI can deliver. Comparing the SVI and VI lines, over much of the plot’s range, OVI thus cannot take less than half the runtime of SVI without outperforming VI itself.

**Fig. 8.**
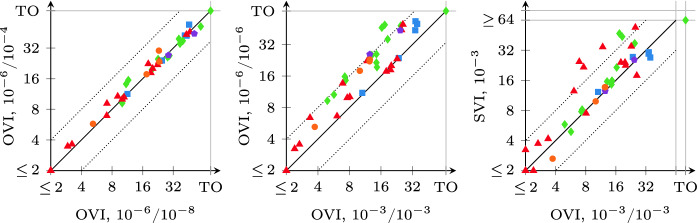
Influence of $\epsilon$/$\alpha$ on runtime (expected rewards, relative error)

**Fig. 9.**
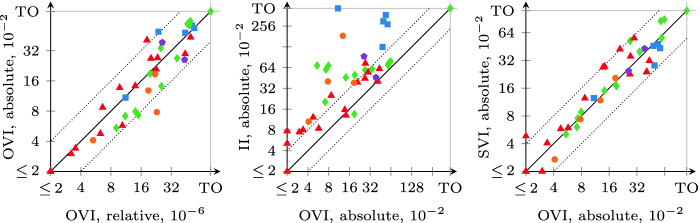
Runtime comparison with absolute error (expected rewards)

### On the Effect of $\epsilon$ and $\alpha$

We also compared the four algorithms for different values of $\epsilon$ and, where applicable, $\alpha$. We show a selection of the results in Fig. 8. The axis labels are of the form “algorithm, $\epsilon$/$\alpha$”. On the left, we see that the runtime of OVI changes if we set $\alpha$ to values different from $\epsilon$, however there is no clear trend: some instances are checked faster, some slower. We obtained similar plots for other combinations of $\alpha$ values, with only a slight tendency towards longer runtimes as $\alpha > \epsilon$. mcsta thus uses $\alpha = \epsilon$ as a default that can be changed by the user.

In the middle, we study the impact of reducing the desired precision by setting $\epsilon$ to $10^{-3}$. This allows OVI to speed up by factors mostly between 1 and 2; the same comparison for SVI and II resulted in similar plots, however VI was able to more consistently achieve higher speedups. When we compare the right plot with the right-hand plot of Fig. 6, we consequently see that the overall result of our comparison between OVI and SVI does not change significantly with the lower precision, although OVI does gain slightly more than SVI.

### Comparing Relative and Absolute Error

In Fig. 9, we show comparison plots for the runtime when using  instead of . Requiring absolute-error-correct results may make instances with low result values much easier and instances with high results much harder. We chose $\epsilon = 10^{-2}$ as a compromise, and the leftmost plot confirms that we indeed chose an $\epsilon$ that keeps the expected-reward benchmarks on average roughly as hard as with $10^{-6}$ relative error. In the middle and right plots, we again see OVI compared with II and SVI. Compared to Fig. 6, both II and SVI gain a little, but there are no significant differences overall. Our experiments thus confirm that the relative performance of OVI is stable under varying precision requirements.

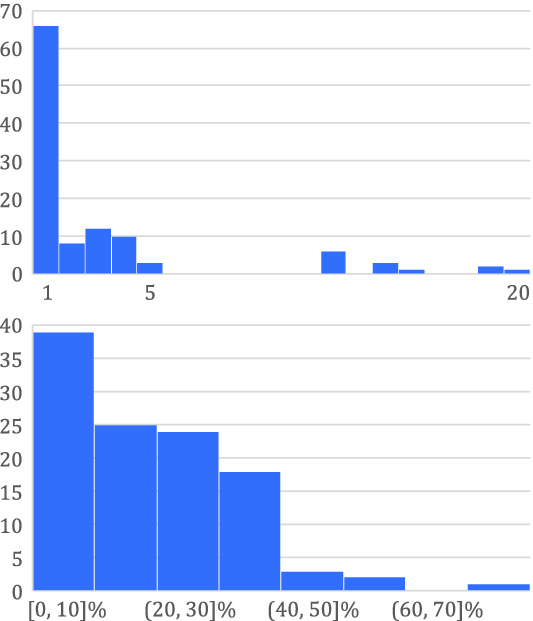



### Verification Phases

On the right, we show histograms of the number of verification phases started (top, from 1 phase on the left to 20 on the right) and the percentage of iterations that are done in verification phases (bottom) over all benchmark instances (probabilities and rewards). We see that, in the vast majority of cases, we need few verification attempts, with many succeeding in the first attempt, and most iterations are performed in the iteration phases.

## Conclusion

We have presented *optimistic value iteration* (OVI), a new approach to making non-exact probabilistic model checking via iterative numeric algorithms sound in the sense of delivering results within a prescribed interval around the true value (modulo floating-point and implementation errors). Compared to interval (II) and sound value iteration (SVI), OVI has slightly stronger termination guarantees in presence of multiple fixed points, and works in practice for max. probabilities without collapsing end components despite the lack of a guarantee. Like II, it can be combined with alternative methods for dealing with end components such as the new *deflating* technique of [23]. OVI is a *simple* algorithm that is *easy* to add to any tool that already implements value iteration, and it is *fast*, further closing the performance gap between VI and sound methods.

## Data Availability

A dataset to replicate our experimental evaluation is archived and available at DOI 10.4121/uuid:3df859e6-edc6-4e2d-92f3-93e478bbe8dc [19].

## Footnotes

1 This can be done via Algs. 2 (for $S_\mathrm {min}^1$) and 4 (for $S_\mathrm {max}^1$) of [12], respectively. These algorithms do not consider the probabilities, but only whether there is a transition and branch (with positive probability) from one state to another or not. We thus call them *graph-based* algorithms, as opposed to *numeric* algorithms like VI itself.
